# Multi-omics analysis identifies stemness-driven molecular subtypes, prognostic signature, epigenetic target APCDD1, and drug candidate Leflunomide in Wilms tumor

**DOI:** 10.3389/fonc.2026.1775326

**Published:** 2026-05-25

**Authors:** Huifang Du, Yifeng Zheng, Chentao Zhu, Yang Guo, Pengfei Li, Peng Hong

**Affiliations:** 1 Huzhou Central Hospital, Fifth School of Clinical Medicine of Zhejiang Chinese Medical University, Zhejiang, China; 2 Affiliated Huishan Hospital of Xinglin College, Nantong University, Wuxi Huishan District People’s Hospital, Wuxi, Jiangsu, China; 3 Chongqing Medical University, Chongqing, China

**Keywords:** APCDD1, Leflunomide, molecular subtypes, prognostic signature, stemness, Wilms tumor

## Abstract

Wilms tumor (WT), the most common pediatric renal malignancy, continues to present therapeutic challenges in refractory and relapsed patients. Stemness plays a crucial role in the development and progression of WT, yet the specific mechanisms involved are not yet fully understood. This study systematically investigated the molecular basis of stemness features in WT using multi-omics data and computational biology approaches. Single-cell RNA sequencing combined with the CytoTRACE algorithm revealed that the blastemal cells exhibited the highest stemness score, which correlated significantly with worse prognosis in WT patients. Consensus clustering based on prognostic stemness-related genes stratified WT samples into two distinct molecular subtypes (C1 and C2). The C1 subtype exhibited higher stemness, an immunosuppressive tumor microenvironment characterized by dysfunctional NK cells, and significantly worse clinical outcomes. We subsequently developed and validated a robust prognostic risk signature using Lasso-Cox regression. Mechanistic investigations uncovered that the tumor-suppressive effect of *APCDD1* was mediated by promoter hypermethylation, and its expression could be restored by the demethylating agent Decitabine. Two-sample Mendelian randomization analysis indicated that elevated *APCDD1* expression had a protective causal effect on WT susceptibility. Through integrative drug repositioning analysis, Leflunomide was identified as a promising candidate for high-risk WT patients. *In vitro* functional assays suggested that Leflunomide potently inhibited the proliferation, migration, and invasion of WT cells, and induced apoptosis in a dose-dependent manner, as validated by CCK-8, EdU, wound healing and Transwell assays, and flow cytometry with Annexin V/PI staining, respectively. In conclusion, this study proposes a preliminary discovery framework for the stemness-driven molecular landscape for WT, defining relevant subtypes, a prognostic signature, and an epigenetic regulator (*APCDD1*). We further propose Leflunomide as a novel therapeutic strategy for high-risk WT patients. These findings advance our understanding of WT biology and provide actionable insights for risk stratification and targeted intervention.

## Introduction

Nephroblastoma, commonly known as Wilms tumor (WT), is the most common malignant renal tumor in the pediatric population and accounts for over 90% of kidney tumors in children under 15 years of age (1–3). In recent years, with continuous refinement of multidisciplinary treatment strategies, including standardized nephrectomy, individualized chemotherapy, and rational application of adjuvant radiotherapy and immunotherapy, the 5-year overall survival rate of certain WT subtypes patients has significantly improved to over 90% (4–6). Nevertheless, approximately 10–15% of WT patients experience relapse or develop refractory disease, among whom the long-term survival rate remains below 50% (7). Moreover, a considerable proportion of long-term survivors suffer from severe late adverse effects, such as bone marrow suppression, renal dysfunction, cardiotoxicity, and secondary malignancies, which substantially compromise their quality of life (4, 8–10). Therefore, elucidating the molecular mechanisms underlying WT pathogenesis, identifying key diagnostic and therapeutic targets, and establishing a precise risk stratification system is crucial for further improving treatment efficacy, reducing therapy-related toxicity, and enhancing long-term outcomes in WT patients.

From the perspective of histogenesis, WT is thought to originate from progenitor cells that undergo developmental arrest or aberrant differentiation during kidney formation (11–13). Its hallmark histopathological feature is the “triphasic pattern” comprising blastemal, epithelial (forming primitive tubule or glomerulus-like structures), and stromal components (14, 15). This embryonic tissue architecture not only reflects a close relationship between WT and normal renal development, but also implies the presence of cancer stem cells (CSCs) with multi-lineage differentiation potential within the tumor (12). At the molecular genetic level, inactivation of the *WT1* gene represents one of the earliest identified pathogenic events, occurring in approximately 10–15% of sporadic WT cases (16). *WT1* encodes a key zinc-finger transcription regulator involved in cell differentiation and apoptosis during kidney development (17, 18). Loss of *WT1* function leads to blocked differentiation of renal progenitor cells, thereby promoting tumorigenesis (19). Furthermore, *WT1* abnormalities often cooperate with aberrant activation of the WNT/β-catenin signaling pathway (20). The latter facilitates nuclear translocation of β-catenin and upregulates downstream target genes such as *MYC* and *CCND1*, thereby enhancing tumor cell proliferation and stemness maintenance (21). Beyond WT1, mutations in other genes including *CTNNB1* (encoding β-catenin), *WTX*, and *TP53* have also been identified in various WT subtypes (16). Collectively, these genetic alterations form a complex regulatory network that profoundly influences tumor heterogeneity and stemness in WT.

The emergence of the CSCs theory in recent years has provided new perspectives for understanding the pathogenesis of WT. CSCs are defined as a small subset of cells within tumors possessing self-renewal capacity, multilineage differentiation potential, and the ability to drive tumorigenesis (22, 23). Research indicates that these cells can not only maintain their own population through asymmetric division but also generate the bulk of tumor cells, serving as a major source of tumor heterogeneity (22, 24). In WT, cell populations expressing renal progenitor markers such as *SIX2* and *CITED1* have been confirmed to exhibit CSC characteristics, capable of reconstructing tumor tissues with typical triphasic patterns both *in vivo* and *in vitro* (24, 25). CSCs often show high expression of surface markers including *CD133*, *CD44*, and *ALDH1*, and rely on signaling pathways such as Wnt/β-catenin, Hedgehog, and Notch to maintain their stemness (22). More importantly, CSCs demonstrate significant resistance to chemotherapy and radiotherapy, attributed to their enhanced DNA damage repair capacity, elevated expression of drug efflux transporters (e.g., ABC transporters), and a predominantly quiescent cell cycle state (22, 26, 27). Consequently, CSCs are considered a critical cellular underpinning for chemoresistance, immune evasion, and recurrence in WT.

While the existence of certain biological features of CSCs in WT have been preliminarily recognized, their specific mechanisms in regulating the tumor microenvironment (TME), metabolic reprogramming, and epigenetic modifications remain incompletely understood (24, 28). Moreover, their translational value for clinical subtyping, prognostic assessment, and treatment selection warrants further exploration. With the advancement of cutting-edge technologies such as single-cell sequencing and spatial transcriptomics, we are now equipped to systematically decipher the cellular heterogeneity, stemness characteristics, and regulatory networks of WT at an unprecedented resolution.

This study systematically deciphered the molecular basis and clinical significance of stemness features in WT through integrated multi-omics data and computational biology approaches. Findings revealed that blastemal cells exhibited the highest stemness scores, which were significantly associated with poorer overall and progression-free survival in WT patients. Utilizing consensus clustering, WT samples were stratified into two subtypes, C1 and C2, with the C1 subtype demonstrating higher stemness scores, inferior prognosis, and an immunosuppressive tumor microenvironment characterized by impaired NK cell function. A risk signature was constructed via Lasso-Cox regression and demonstrated robust prognostic performance across both training and validation cohorts. Interpretable SHAP analysis further identified *APCDD1* as the feature with the highest contribution within the prognosis signature, where its elevated expression correlated significantly with favorable outcomes. Two-sample Mendelian randomization analysis provided evidence that increased expression of *APCDD1* and *BICC1* was causally associated with a reduced risk of WT, suggesting potential protective roles for these genes. Mechanistic investigations indicated that *APCDD1* expression was suppressed due to hypermethylation of its promoter region. Additionally, drug repositioning analysis identified Leflunomide as a candidate therapeutic drug, potentially targeting molecules such as *IL20RA* and *HOXB1* to modulate stemness-associated genes, thereby exhibiting antitumor effects particularly in C1-subtype/high-risk WT patients.

**Figure 1:**
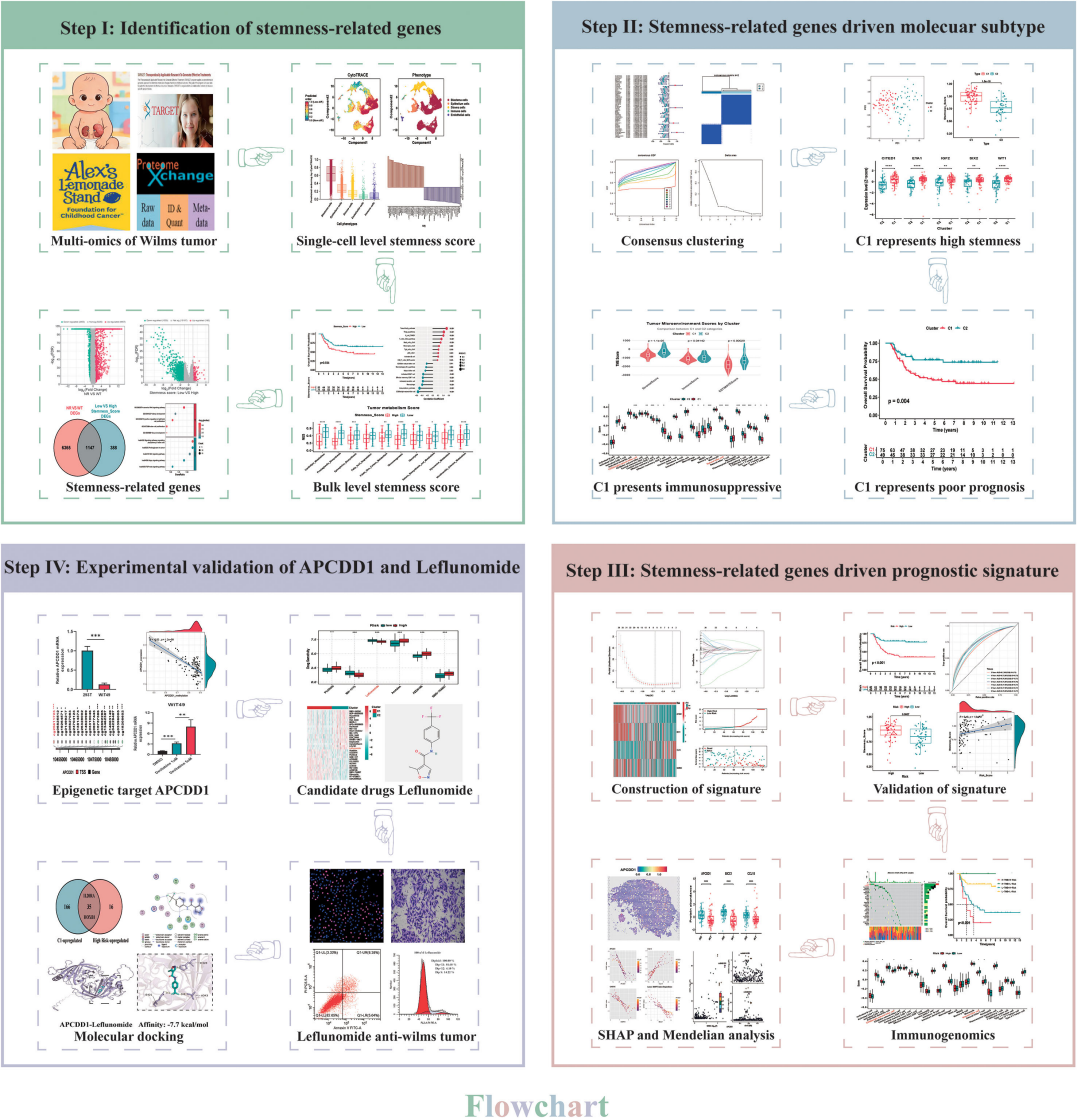
Technical roadmap of this research.

To address the challenges in understanding and targeting CSCs in WT, this study employed a multi-omics approach to systematically decode the stemness landscape. We defined distinct molecular subtypes, developed a prognostic signature, and identified *APCDD1* as a key protective gene with a deregulated epigenetic mechanism. Furthermore, we proposed Leflunomide as a candidate for drug repurposing. A schematic overview of the study design and workflow is presented in **Figure 1**. Collectively, these findings provide a framework for advancing precision medicine in WT, offering new strategies for prognosis prediction and targeted therapy.

## Materials and methods

### Data sources

RNA-sequencing data and corresponding complete survival information for 124 WT samples were obtained from the TARGET-WT database (29). Single-cell RNA-seq data from 6 WT samples and spatial transcriptomic data from 2 WT samples were obtained from the Single-cell Pediatric Cancer Atlas (30). Mass spectrometry-based proteomics data for 88 WT samples and 71 matched adjacent renal tissues were provided by ProteomeXchange (14). Additionally, RNA-seq data for 62 WT samples and 37 adjacent renal tissues were retrieved from the Genomic Sequence Archive for Human (14). Outcome data for WT were obtained from the FinnGen R12 cohort, using the case definition specified by the “finngen_R12_C3_WILMS_TUMOR” field. Exposure eQTL data for the two-sample Mendelian randomization analysis were acquired from the public release of the eQTL Catalogue. All data used in this study were de-identified and publicly available, having been obtained in accordance with the respective data access agreements. Therefore, no additional ethical approval was required for this study. The procedures for raw data processing, quality control, and analysis strictly followed the corresponding official documentation and previously published methodologies.

### Identification of stemness-related gene set

Based on the quality-controlled and cell-type-annotated Seurat object, the RNA expression matrix (using normalized log-transformed counts as input) was extracted, retaining only genes detected in at least five cells to minimize interference from low-abundance genes in downstream analyses. The filtered expression matrix was then input into CytoTRACE v1.0 with default parameters to calculate a stemness score for each cell, ranging from 0 to 1, where a higher score indicates higher stemness and lower differentiation potential (31). Subsequently, the stemness scores were averaged within each cell subpopulation based on their annotation labels. Spearman correlation analysis was performed between the expression of each gene and the stemness score of its corresponding cell. Genes significantly associated with stemness were identified using multiple comparison correction (FDR < 0.05 and correlation coefficient ρ ≥ 0.3), forming a “Stemness-related Gene Set”.

### Consensus clustering

Consensus clustering is an unsupervised learning framework used to assess clusters stability and determine the optimal number of clusters (k) and others parameter. This is achieved by performing multiple bootstrap resampling iterations, each followed by clustering of the sampled subset (32). The core analysis in this study employed the K-means algorithm as the base clusterer with Euclidean distance as the metric. For each iteration, 80% of the samples were drawn via bootstrap sampling, and this process was repeated independently 50 times. The results from these 50 iterations were aggregated to construct a consensus matrix, where each element represents the frequency with which any two samples were assigned to the same cluster across all iterations. Subsequently, hierarchical clustering was applied to the consensus matrix. The optimal number of clusters (k) was selected from a candidate range by evaluating stability metrics, primarily based on the empirical cumulative distribution function of the consensus indices.

### Construction and validation of the prognostic signature

The 124 WT samples were randomly divided into training set and test set at a 1:1 ratio for the development and independent validation of a multi-gene prognostic signature based on gene expression. In the training set, high-dimensional gene expression data were first standardized. Lasso-Cox regression was then employed for feature selection. The optimal regularization parameter (λ) was determined through 10-fold cross-validation, resulting in a sparse set of features with enhanced generalizability (33). Subsequently, a multivariate Cox proportional hazards regression was fitted in the training set using the retained features to estimate their regression coefficients. A risk score for each sample was calculated as the weighted sum of the expression levels of these features multiplied by their respective coefficients. Samples were dichotomized into low-risk and high-risk groups using the median risk score of the training set as the cutoff. This same threshold was applied to the test set. For survival analysis, Kaplan-Meier survival curves were generated for the two groups, and the statistical significance of the difference was assessed using the log-rank test. Time-dependent receiver operating characteristic (ROC) analysis was performed at pre-specified time points to evaluate the signature’s predictive accuracy, with the corresponding area under the curve calculated. Furthermore, correlation analyses were conducted to investigate the independence of the risk score and its relationship with other clinical variables.

### *In silico* drug sensitivity prediction and screening

Drug sensitivity was predicted across datasets using the oncoPredict package (34). During the training phase, gene expression profiles from the GDSC2 database, along with their corresponding drug response data, were used as the training set. The trained predictor was subsequently applied to the independent transcriptomic dataset to generate a sensitivity score for each sample-drug pair. These scores were log-transformed to stabilize variance. For downstream analysis, the predicted phenotypes were integrated with clinical risk information to form grouping variables. Non-parametric tests were used to compare drug sensitivity differences between risk groups, identifying candidate drugs under a statistically significant framework. A secondary screening of candidate drugs was performed using the Connectivity Map (CMap) database (35). Differentially expressed genes were mapped to the CMap, and the CMap query tool was employed to obtain connectivity scores for each drug. Drugs exhibiting a negative connectivity score with the given transcriptomic signature were prioritized, as this indicates their potential to reverse the signature.

### Cell lines and drug intervention

The Wilms tumor cell line WiT49 and the human embryonic kidney cell 293T were cultured in DMEM supplemented with 10% fetal bovine serum and 1% penicillin-streptomycin at 37 °C in a humidified atmosphere containing 5% CO_2_. Leflunomide (MedChemExpress, HY-B0063) was dissolved in DMSO to prepare a 10 mM stock solution, which was stored protected from light at -80 °C. Prior to experiments, the stock solution was diluted to the desired working concentrations using complete culture medium, ensuring a final DMSO concentration of less than 0.1%. Decitabine (MedChemExpress, HY-A0004) was similarly reconstituted in DMSO. Cells were seeded at densities of 5×10³ cells/well in 96-well plates, 2×10^5^ cells/well in 6-well plates, or 1×10^6^ cells/dish in 10 cm dishes. After allowing 12 hours for attachment, the cells were treated with drugs. The treatment duration and specific conditions were tailored to the requirements of the respective downstream assays. Following drug treatment, cell viability was assessed using the CCK-8 assay kit (MedChemExpress, HY-K0301). Briefly, CCK-8 reagent was added to the culture medium, followed by incubation at 37 °C for 2 hours. The absorbance at a wavelength of 450 nm was then measured using a microplate reader.

### Functional assays

Following drug intervention, WiT49 cell proliferation was assessed using the EdU assay kit (Beyotime, C0075S). Cells were labeled with EdU working solution for 2 hours, fixed with 4% paraformaldehyde, and subjected to fluorescence staining. Alexa Fluor 555 azide was used for EdU detection, and nuclei were counterstained with DAPI. Images were captured randomly using a fluorescence microscope. The EdU-positive rate was calculated as the ratio of EdU-positive cells to DAPI-positive cells. For cell cycle analysis, cells treated with drugs for 48 hours were trypsinized, washed twice with phosphate-buffered saline, and fixed in 70% ethanol overnight at 4 °C. The cell cycle distribution (proportions in G0/G1, S, and G2/M phases) was analyzed by flow cytometry, and the data were fitted using ModFit LT 5.0 software. Apoptosis was evaluated by Annexin V-FITC/PI double staining after 48 hours of drug intervention. Cells were analyzed by flow cytometry, and the percentages of early and late apoptotic cells (Annexin V^+^/PI^-^ and Annexin V^+^/PI^+^, respectively) were quantified using FlowJo software. The wound healing assay was performed to assess cell migration. When cells reached approximately 90% confluence, a uniform scratch was created in the monolayer using a sterile 10 μL pipette tip. Floating cells were removed by washing with PBS, and the culture medium was replaced with serum-free medium containing the respective drugs. Images of the wound area were captured at 0 and 48 hours under a microscope. For the Transwell assay, cell migration and invasion were evaluated. Chambers with 8 μm pores were used for the migration assay. For the invasion assay, the chambers were pre-coated with Matrigel. A cell suspension in serum-free medium containing drugs was added to the upper chamber, while the lower chamber was filled with complete medium containing 20% fetal bovine serum as a chemoattractant. After 48 hours of incubation, cells that had migrated or invaded to the lower surface of the membrane were fixed with 4% paraformaldehyde, stained with 0.1% crystal violet, and non-migrated/invaded cells on the upper surface were carefully removed with a cotton swab. The number of cells in randomly selected fields was counted under a microscope.

### Statistical analysis

All experiments were independently repeated at least three times. Data are presented as the mean ± standard deviation. Comparisons among multiple groups were performed using one-way analysis of variance. *P* < 0.05 was considered statistically significant. For all correlation and multi-group analyses, multiple test corrections were performed using the Benjamini-Hochberg method. All statistical analyses and graph generation were conducted using GraphPad Prism software (version 9.5) and the R-Project (version 4.3.1). Key bioinformatics analyses were implemented using specific R packages: ConsensusClusterPlus was used for molecular subtyping and stability assessment, glmnet was employed for Lasso-Cox regression and feature selection, and fastshap was utilized for calculating SHAP values to interpret model contributions.

## Results

### Analysis of stemness characteristics in Wilms tumor based on CytoTRACE

**Figure 2:**
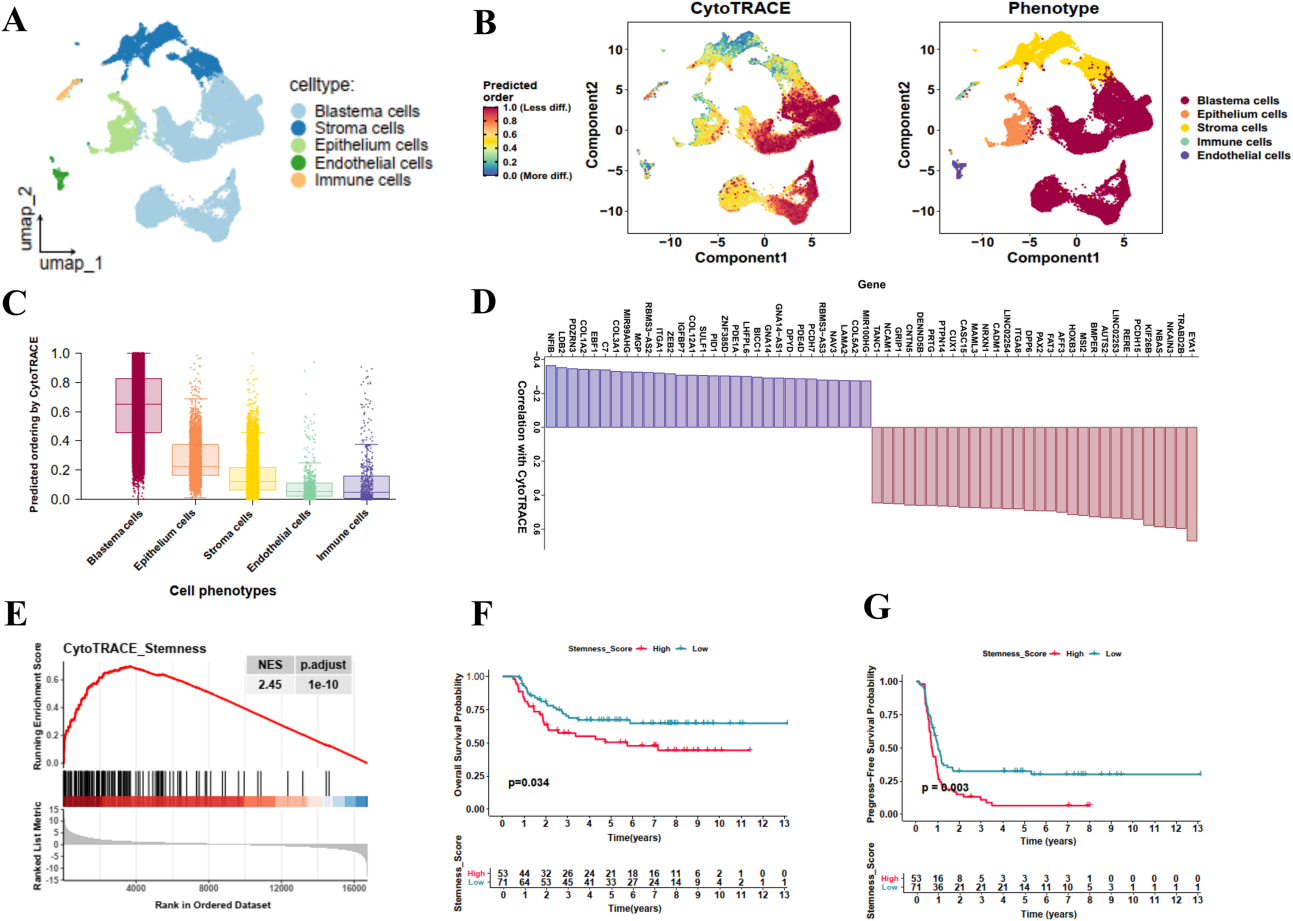
CytoTRACE algorithm delineates the stemness landscape of Wilms tumor. **(A)** UMAP visualization of single-cell RNA sequencing data reveals the cellular landscape across 6 Wilms tumor samples, colored by 5 distinct cell types. **(B)** CytoTRACE algorithm quantify differentiation potential across cell phenotypes, **(C)** with blastema cells showing the highest stemness, while immune and endothelial cells show the lowest. **(D)** Correlation analysis of the top 100 genes with CytoTRACE scores, with red indicating positive correlation and blue indicating negative correlation. **(E)** Gene Set Enrichment Analysis results for the gene set associated with CytoTRACE scores. Wilms tumor patients with higher stemness-score exhibited significantly lower **(F)** overall and **(G)** progression-free survival.

To gain deeper insights into cellular heterogeneity and stemness potential within the WT microenvironment, we performed high-resolution single-cell transcriptomic analysis on WT tissue samples. This analysis systematically classified the cells into five major types: blastemal, stromal, epithelial, endothelial, and immune cells (**Figure 2A**), effectively recapitulating the classic triphasic histological pattern of WT. Subsequently, we applied the CytoTRACE algorithm to assess the stemness state of each cell type (**Figure 2B**). The results revealed that blastemal cells exhibited a significantly higher average CytoTRACE score compared to the other four cell types (**Figure 2C**), indicating their superior stemness potential. We further calculated the Pearson correlation coefficient between the expression of all detectable genes and the CytoTRACE score across the single cells (**Figure 2D**). Using a correlation coefficient threshold of ≥ 0.3, we identified 149 genes highly associated with WT stemness (**Supplementary File 1**). This gene set included known stemness markers such as *EYA1* and *NCAM1*, alongside several novel stemness-associated genes identified for the first time in WT. The majority of these genes showed a significant correlation with patient prognosis (**Supplementary Figure 1A**), suggesting their potential critical roles in maintaining the stemness of blastemal cells and driving WT progression. Gene set enrichment analysis conducted on this gene set revealed a significant positive association with WT tumorigenesis (**Figure 2E**). Furthermore, we computed a “stemness score” based on this gene set for 124 WT patient samples and stratified the patients into high and low stemness score groups. Patients in the high stemness score group demonstrated significantly worse overall survival and progression-free survival compared to those in the low stemness score group (**Figures 2F, G**; **Supplementary Figures 1B–E**). These findings reinforce the potential role of the stemness in WT tumorigenesis and progression, and provide a foundation for further elucidating the biological distinctions between WT patients with high versus low stemness characteristics.

### Identification of stemness-related genes

**Figure 3:**
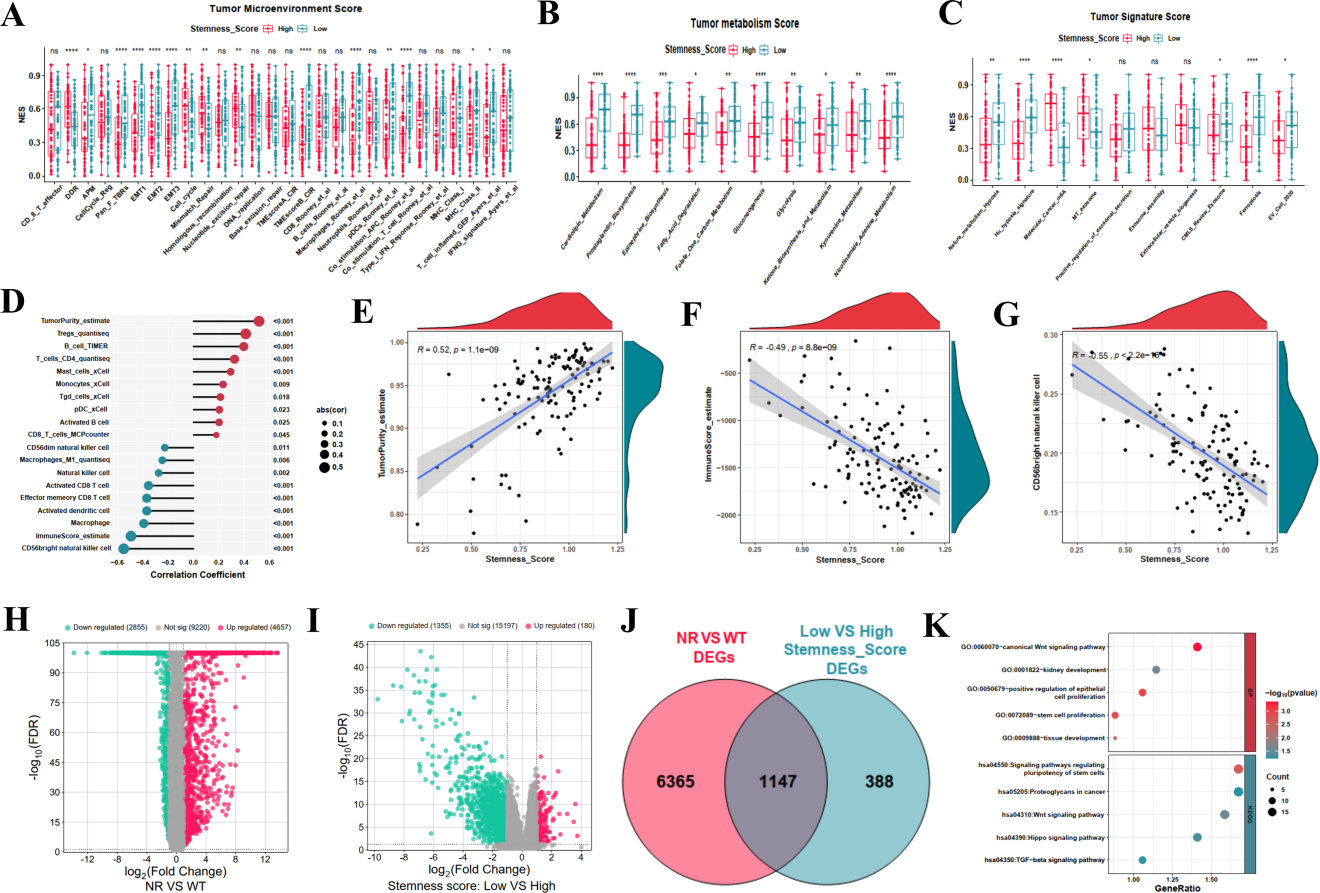
Characterization of the stemness-related phenotype in Wilms tumor. **(A)** Comparison of tumor microenvironment scores between high-stemness and low-stemness groups. **(B)** Metabolic pathway enrichment analysis reveals globally reduced metabolic activity in the high-stemness group. **(C)** Hallmark gene set analysis revealed that the high-stemness group had lower ferroptosis and hypoxia pathway scores but a higher m6A modification score. **(D)** Correlation heatmap depicting the associations between the stemness score and the infiltration levels of various immune cell types. Scatter plots demonstrating significant correlations of the stemness score with tumor purity **(E)**, immune score **(F)**, and CD56bright NK cell infiltration **(G)**. Volcano plot showing differentially expressed genes between **(H)** tumor and adjacent non-tumor tissues, and **(I)** high-stemness and low-stemness groups. **(J)** Venn diagram showing the overlap of differentially expressed genes between high-stemness and low-stemness groups with those between tumor and adjacent non-tumor tissues. **(K)** Functional enrichment analysis of the 1147 overlapping genes (defined as stemness-related genes).

We compared the tumor microenvironment, metabolic characteristics, and gene set enrichment related to malignant phenotypes between WT patients with high and low stemness scores. Results showed that the high stemness group had a significantly lower immune microenvironment score compared to the low stemness group. Furthermore, activity of the EMT-related gene set was significantly decreased, while activity of the DNA damage response-related gene set was significantly upregulated in the high stemness group (**Figure 3A**). This suggests that high-stemness WT may maintain stemness by suppressing the stromal-EMT axis and enhancing DNA damage repair, thereby fostering an immunosuppressive and genomically relatively quiescent microenvironment. Metabolic pathway enrichment was significantly lower in the high stemness group compared to the low stemness group (**Figure 3B**). Specifically, the high stemness group exhibited significantly lower scores for ferroptosis and hypoxia-related pathways, but a significantly higher m6A modification score (**Figure 3C**). Significant correlations were observed between the stemness score and the infiltration levels of various immune cells (**Figure 3D**). The stemness score was positively correlated with tumor purity and regulatory T cell infiltration (**Figure 3E**), but negatively correlated with immune score and CD56bright NK cells infiltration (**Figures 3F, G**). Collectively, high-stemness WT exhibits characteristics of an immunosuppressive microenvironment, low metabolic activity, reduced ferroptosis/hypoxia response, and elevated m6A levels. This profile suggests a quiescent, genomically relatively stable tumor microenvironment centered around stemness maintenance.

A comparative analysis of gene expression between high and low stemness score WT patients identified 1535 differentially expressed genes (DEGs) (**Figures 3H, I**). Among these, 1147 DEGs were also significantly differentially expressed between WT tumor tissues and adjacent non-tumor tissues (**Figure 3J**). Functional enrichment analysis of these 1147 overlapping DEGs revealed their primary involvement in biological processes such as the WNT signaling pathway, kidney development, and stem cell proliferation (**Figure 3K**). Based on their common differential expression patterns, these 1147 genes were defined as WT stemness-related genes (SRGs). This gene set aims to provide a molecular foundation for elucidating WT stemness maintenance mechanisms, understanding WT heterogeneity, and developing related targeted therapeutic strategies.

### Stemness-related genes driven molecular subtype of Wilms tumor

**Figure 4:**
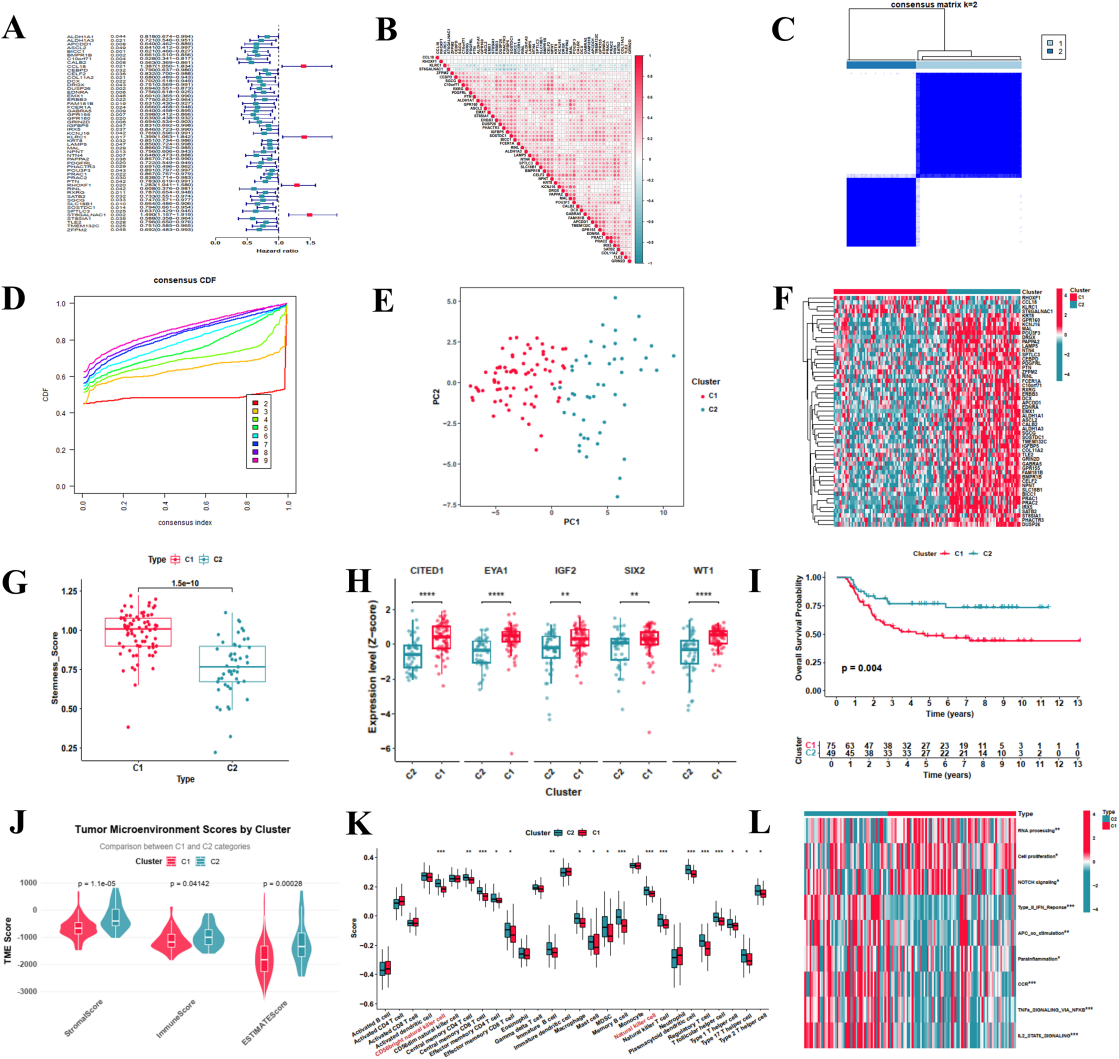
Identification and characterization of a stemness-related molecular subtype in Wilms tumor. **(A)** Cox proportional hazards regression analysis identified 53 stemness-related genes (SRGs) significantly associated with patient overall survival. **(B)** Co-expression correlations among the 53 prognostic SRGs. **(C, D)** Consensus clustering based on these 53 prognostic SRGs defined two robust molecular subtypes Wilms tumor. **(E)** Principal component analysis confirming the transcriptomic distinction between C1 and C2 subtypes. **(F)** Heatmap of 53 prognostic SRGs expression across subtypes. **(G)** Comparison of stemness score between C1 and C2 subtypes. **(H)** Comparison of classical stemness marker expression between C1 and C2 subtypes. **(I)** Kaplan-Meier survival curves for the two subtypes. **(J)** Comparison of immune scores between C1 and C2 subtypes. **(K)** Comparison of the infiltration levels of 28 types of immune cells between C1 and C2 subtypes. **(L)** GSVA highlighting pathway alterations between C1 and C2 subtypes. * P<0.05, ** P<0.01, *** P<0.001.

We first conducted survival analysis on the 1147 SRGs, identifying 53 genes significantly associated with the overall survival of WT patients, most of which exhibited a protective effect (**Figure 4A**). Significant co-expression correlations were observed among these 53 genes, suggesting they constitute a co-regulated module involved in coordinated cellular functions (**Figure 4B**). Consensus clustering based on these 53 prognostic genes robustly classified the WT samples into two distinct molecular subtypes, designated C1 and C2 (**Figures 4C, D**). Principal component analysis confirmed clear separation between the two subtypes across the global transcriptome (**Figure 4E**), and the expression of most SRGs differed significantly between C1 and C2 (**Figure 4F**), revealing substantial molecular heterogeneity of WT.

The C1 subtype was characterized by a significantly higher stemness score compared to C2 (**Figure 4G**), accompanied by elevated expression of classical stemness marker genes such as *CITED1*, *WT1* and *SIX2* (**Figure 4H**). The ggalluviall diagram was used to visually illustrate the distribution of the C1 and C2 molecular subtypes across major histological types (**Supplementary Figure 2**). Correspondingly, patients in the C1 subtype had significantly poorer survival outcomes (**Figure 4I**), indicating that enhanced stemness characteristics are associated with an unfavorable prognosis. Furthermore, the C1 subtype demonstrated a lower immune score than C2 (**Figure 4J**), suggesting an immunosuppressive TME. This was supported by significantly reduced infiltration of NK cells and downregulation of NK cell-mediated immune responses (**Figure 4K**), indicating impaired NK-mediated immune surveillance. Gene Set Variation Analysis revealed a systemic suppression of antigen presentation, T cell activation, and inflammatory signaling pathways in C1, consistent with an immunosuppressive phenotype (**Figure 4L**). Conversely, modules related to “stemness and self-renewal,” such as NOTCH signaling and proliferation, were maintained or elevated in C1. In summary, the C1 molecular subtype exhibits a concurrent pattern of immunosuppression and high stemness, which likely cooperatively contribute to its poor prognosis.

### Stemness-related genes driven prognostic signature of Wilms tumor

**Figure 5:**
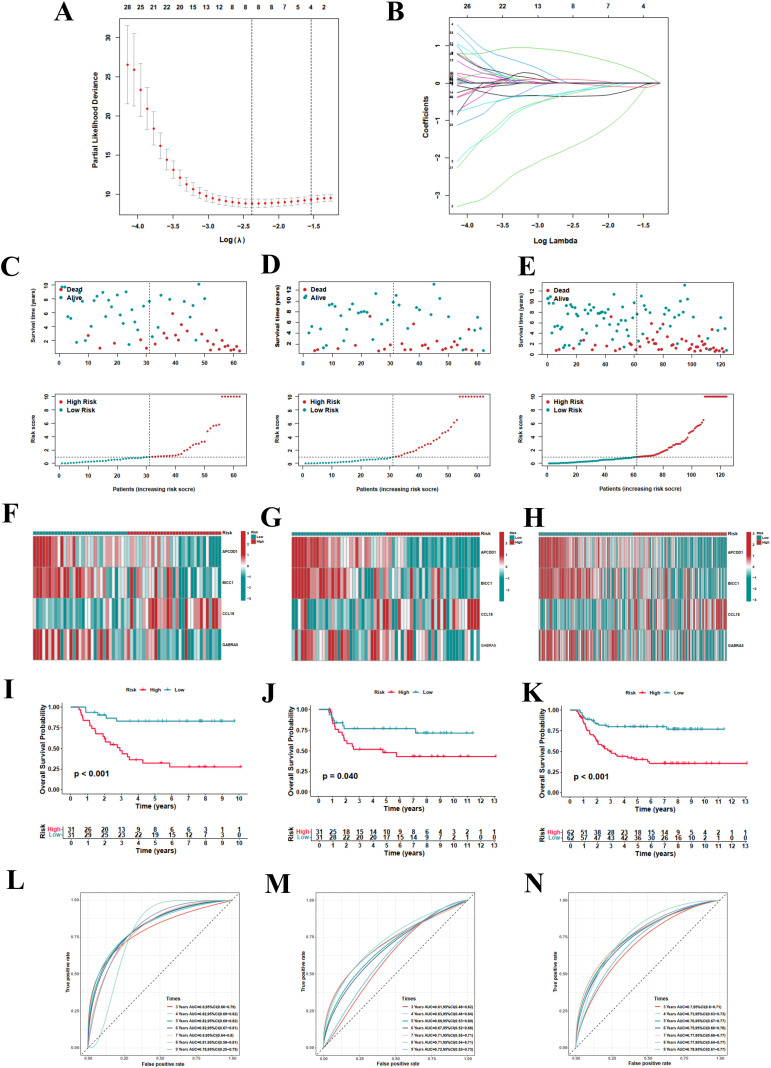
Construction and validation of a stemness-related prognostic signature for Wilms tumor. **(A, B)** *APCDD1*, *BICC1*, *CCL18*, and *GABRA5* were identified as prognostic risk genes for the signature via LASSO Cox regression. Distribution of risk scores, patient survival status, and expression heatmap of the four signature genes in the training **(C, F)**, test **(D, G)**, and entire **(E, H)** cohorts. Kaplan-Meier survival curves demonstrating significantly poorer overall survival in the High-risk group across the training **(I)**, test **(J)**, and entire **(K)** cohorts. Time-dependent ROC curves evaluating the predictive accuracy of the signature at 3–9 years, for the training **(L)**, test **(M)**, and entire **(N)** cohorts.

To evaluate the predictive value of SRGs expression for clinical outcomes, the 124 WT samples were randomly divided into training and test sets. In the training set, LASSO regression was applied to the 53 prognostic SRGs, and the optimal penalty parameter (λ) was determined via 10-fold cross-validation. This process identified four risk genes: *APCDD1*, *BICC1*, *CCL18*, and *GABRA5* (**Figures 5A, B**). Subsequently, a Cox proportional hazards regression signature was fitted in the training set using these four genes to obtain their respective regression coefficients. A multi-gene risk signature and corresponding risk score were constructed for each sample by calculating the weighted sum of the expression levels of these four genes multiplied by their coefficients. Using the median risk score from the training set as a cutoff, WT samples were stratified into low-risk and high-risk groups. The risk score showed a significant positive correlation with mortality events (**Figures 5C–E**). Expression levels of *APCDD1*, *BICC1*, and *GABRA5* were higher in the low-risk group, whereas *CCL18* expression was elevated in the high-risk group (**Figures 5F–H**). Kaplan-Meier analysis revealed that the high-risk group had significantly worse overall survival compared to the low-risk group in both the training and testing sets (**Figures 5I–K**). Time-dependent ROC analysis demonstrated high predictive accuracy of the risk signature at 3 to 9 years (**Figures 5L–N**).

**Figure 6:**
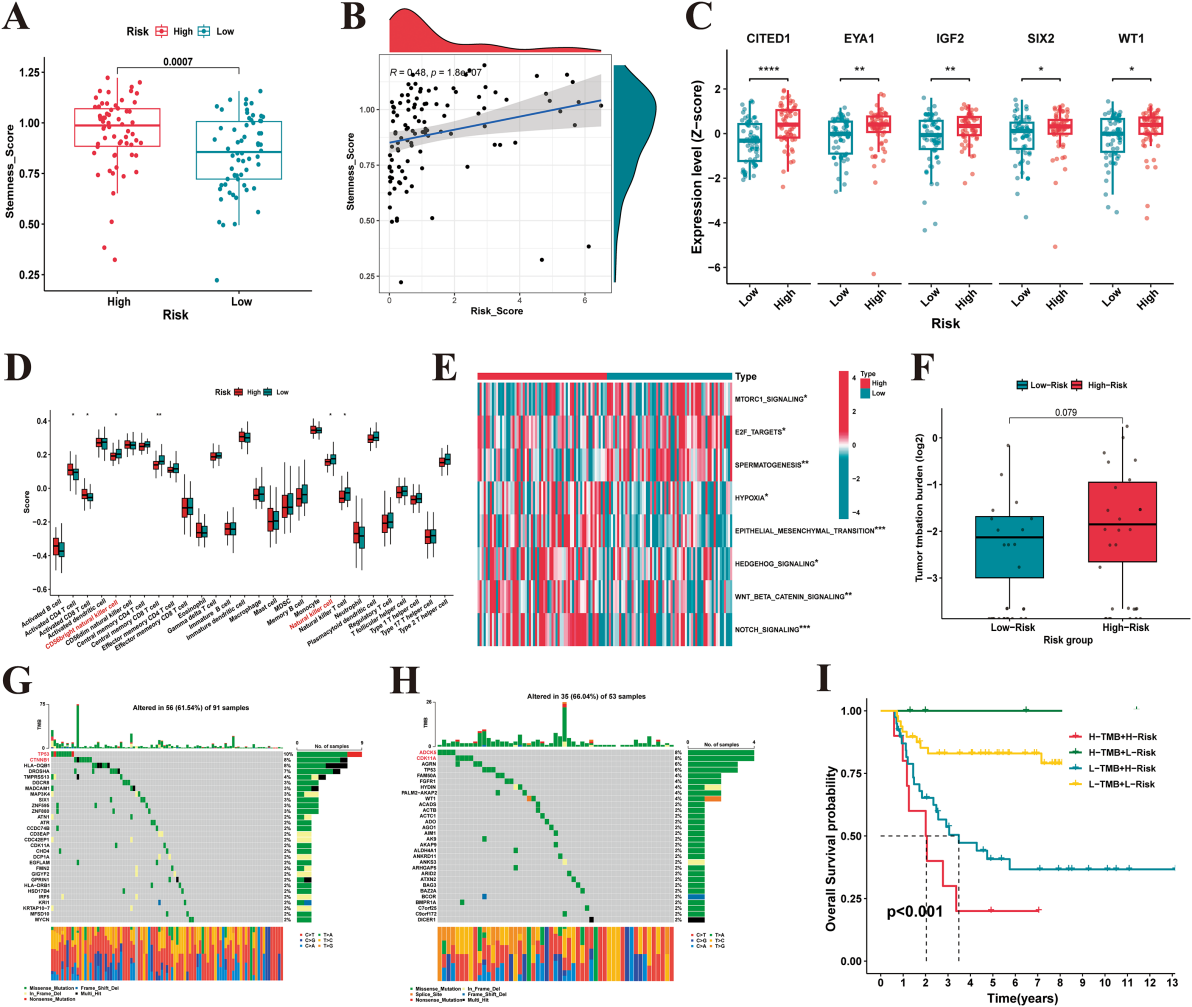
Comparative characterization of High-risk and Low-risk groups in Wilms tumor. **(A)** Comparison of stemness score between High-risk and Low-risk groups. **(B)** Positive correlation between stemness score and risk score. **(C)** Comparison of classical stemness marker expression between High-risk and Low-risk groups. **(D)** Comparison of the infiltration levels of 28 types of immune cells between High-risk and Low-risk groups. **(E)** GSVA highlighting pathway alterations between High-risk and Low-risk groups. **(F)** Comparison of tumor mutational burden between High-risk and Low-risk groups. Mutation profiles are distinct between High-risk **(G)** and Low-risk **(H)** groups. **(I)** Combined analysis of risk score and tumor mutational burden identifies a patient subgroup with the poorest prognosis. * P<0.05, ** P<0.01, **** P<0.0001.

A significant positive correlation was observed between the stemness score and the risk score (**Figures 6A, B**). At the molecular level, high-risk WT samples exhibited upregulation of *WT1*, *SIX2*, and *CITED1*, concomitant with an immunosuppressive microenvironment (**Figures 6C, D**). Furthermore, high-risk samples showed enrichment of core pathways driving proliferation, hypoxia adaptation, and EMT (**Figure 6E**). In contrast, low-risk samples displayed characteristics of activated anti-tumor immunity. Tumor mutational burden (TMB) analysis indicated a higher mutation load in high-risk WT samples (**Figure 6F**), with higher mutation frequencies in *TP53* and *CTNNB1* (**Figure 6G**). Low-risk samples were characterized by mutations in *ADCK5* and *CDK11A* (**Figure 6H**). Combined survival analysis integrating TMB and the risk score showed that patients with both high-risk and high-TMB had the worst prognosis (**Figure 6I**), suggesting that combined assessment may enable more refined risk stratification. In summary, the multi-gene risk signature, derived from SRGs, serves as a reliable tool for prognostic assessment and risk stratification in WT.

### Cross-omics integration, SHAP interpretation, and Mendelian randomization inferences

**Figure 7:**
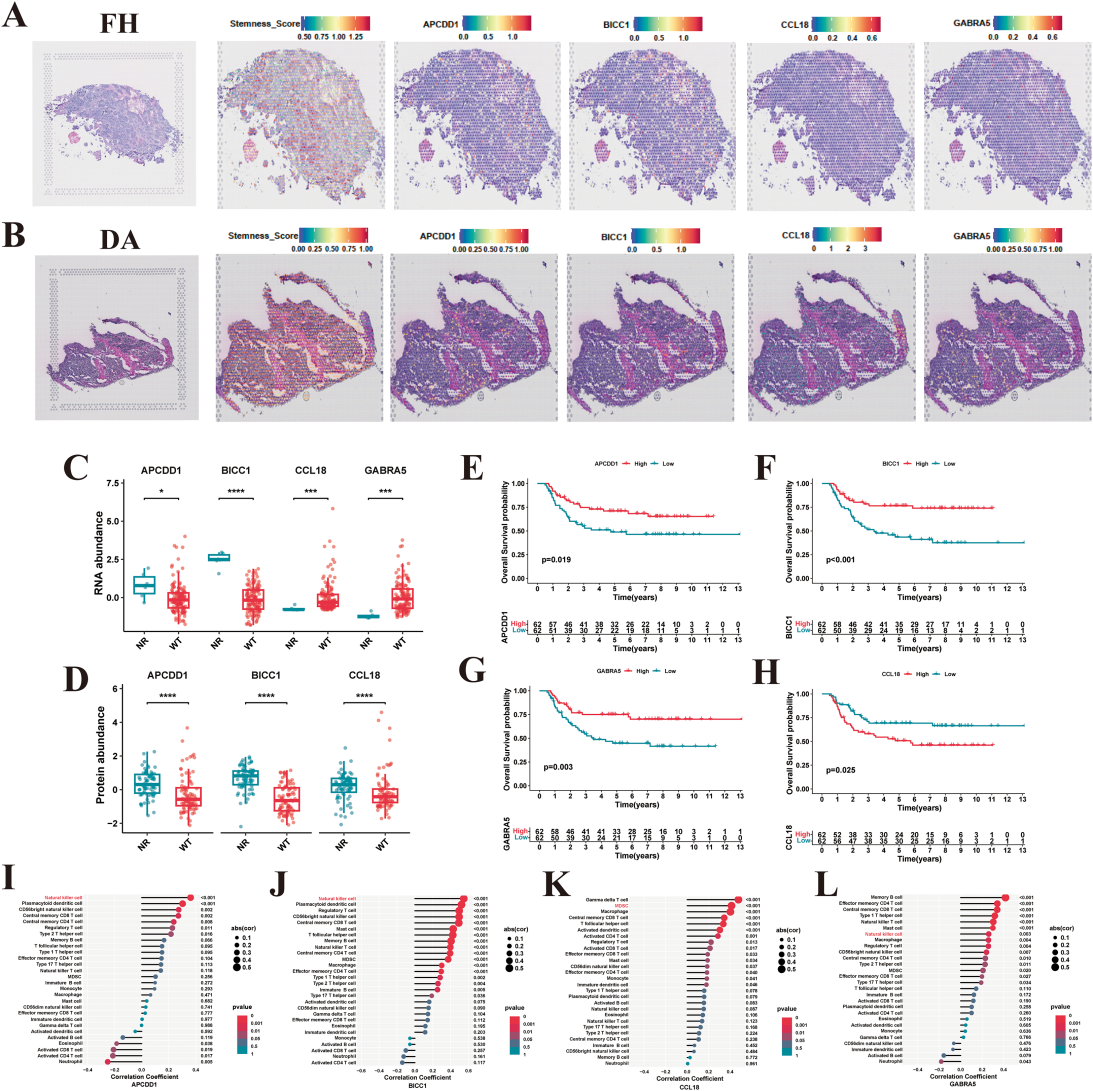
Functional Characterization of risk genes in Wilms tumor. Spatial expression patterns of *APCDD1*, *BICC1*, *GABRA5*, and *CCL18* in FH-WT **(A)** and DA-WT **(B)** subtypes. Validation of significantly reduced mRNA **(C)** and protein **(D)** levels of *APCDD1* and *BICC1* in Wilms Tumor samples compared to adjacent normal kidney tissues. Prognostic impact of risk genes, with *APCDD1* **(E)**, *BICC1* **(F)**, and *GABRA5* **(G)** being favorable factors and *CCL18* **(H)** being a risk factor. Correlations of *APCDD1* **(I)**, *BICC1* **(J)**, *CCL18* **(K)**, and *GABRA5* **(L)** expression with various immune cell infiltration.

Building upon the established risk signature, we systematically analyzed the spatial expression of *APCDD1*, *BICC1*, *GABRA5*, and *CCL18*, and their associations with prognosis and the immune microenvironment using spatial transcriptomic data from the FH-WT and DA-WT subtypes. In FH-WT, the stemness score was significantly elevated in nuclear aggregation regions. Conversely, in non-nuclear aggregation regions, *APCDD1* and *BICC1* expression was enriched, suggesting their role in maintaining low stemness by potentially promoting differentiation and inhibiting proliferation within these local niches. *GABRA5* and *CCL18* exhibited regional expression heterogeneity across both subtypes (**Figures 7A, B**). Compared to adjacent normal kidney tissues, WT samples showed significantly reduced mRNA and protein levels of *APCDD1* and *BICC1* (**Figures 7C, D**). Kaplan-Meier and Cox regression analyses yielded consistent results: high expression of *APCDD1*, *BICC1*, and *GABRA5* was significantly associated with favorable prognosis, whereas high *CCL18* expression correlated with poor prognosis (**Figures 7E–H**). Immune correlation analysis revealed that expression of *APCDD1*, *BICC1*, and *GABRA5* was significantly positively correlated with NK cell infiltration. In contrast, *CCL18* expression showed a significant positive correlation with MDSC infiltration (**Figures 7I–L**). This suggests that these risk genes collectively participate in shaping the WT immune microenvironment.

**Figure 8:**
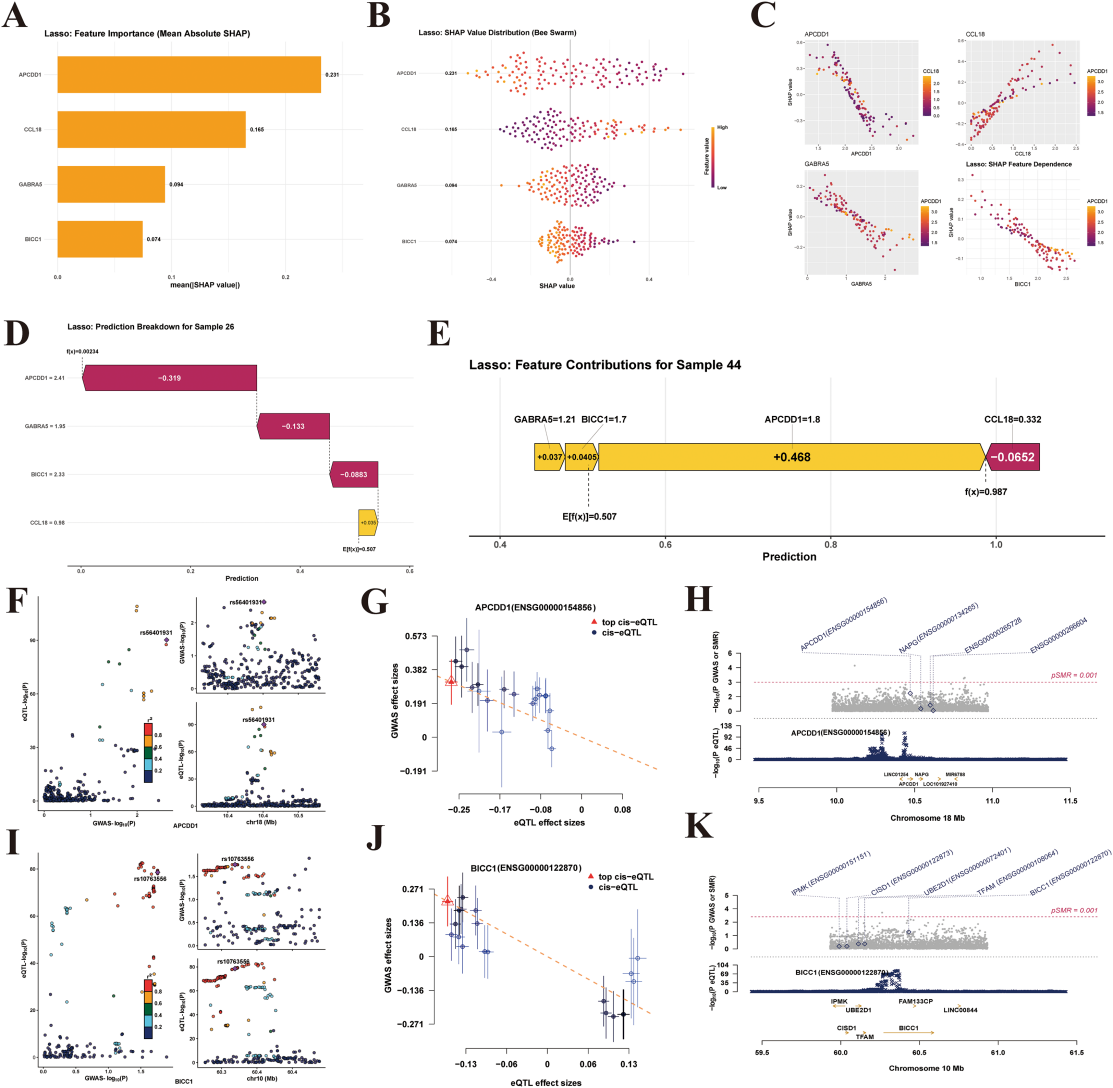
Causal roles and signature contributions of risk genes in Wilms tumor. **(A–C)** Global SHapley Additive exPlanations analysis ranks the feature importance of risk genes, identifying APCDD1 as the top contributor to the prognostic signature. **(D, E)** Local SHapley Additive exPlanations analysis illustrates the direction and magnitude of each gene’s impact on individual predictions. Two-sample Mendelian Randomization analysis reveals a significant protective causal effect of genetically predicted higher *APCDD1* **(F–H)** and *BICC1* **(I–K)** expression on Wilms tumor susceptibility, supported by colocalization evidence.

Global SHapley Additive exPlanations (SHAP) value analysis identified *APCDD1* as having the highest mean absolute SHAP value (**Figures 8A–C**), indicating its dominant contribution to the predictive signature. Local SHAP analysis showed that high *APCDD1* expression consistently pushed the prediction toward a better prognosis for most samples, whereas high *CCL18* expression was associated with a worse prognostic direction. *BICC1* and *GABRA5* provided moderate protective contributions (**Figures 8D, E**). Furthermore, two-sample Mendelian Randomization analysis, using WT susceptibility as the outcome, demonstrated a significant negative causal relationship between genetically predicted higher *APCDD1* expression levels and WT risk, suggesting a protective effect. Colocalization analysis provided evidence for shared causal variants between the genetic signals influencing *APCDD1* expression and those affecting WT susceptibility (**Figures 8F–H**). A similar protective causal trend was observed for *BICC1* (**Figures 8I–K**). Based on these findings, *APCDD1* and *BICC1* emerge as both potential biomarkers and therapeutic targets for WT.

### Promoter hypermethylation mediated transcriptional silencing of APCDD1

**Figure 9:**
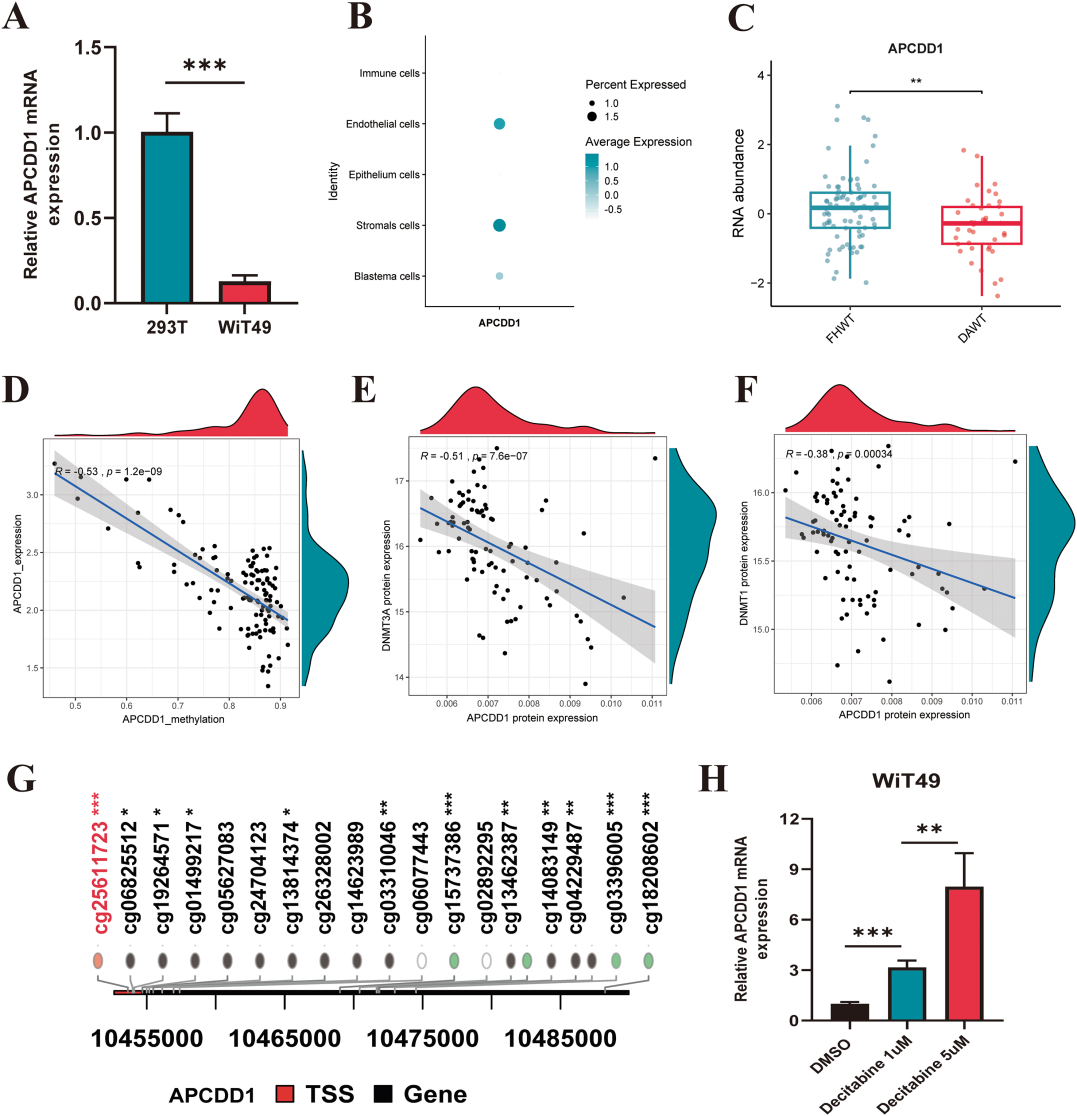
Promoter hypermethylation-mediated transcriptional silencing of *APCDD1* in Wilms tumor. **(A, B)** *APCDD1* mRNA expression is significantly downregulated in the WT cell line WiT49 compared to 293T cells. **(C)** Comparison of *APCDD1* expression between FH-WT and DA-WT. **(D)** Significant negative correlation between *APCDD1* expression and promoter methylation beta value. *APCDD1* expression negatively correlates with the expression of DNA methyltransferases *DNMT3A* **(E)** and *DNMT1* **(F)**. **(G)** Schematic diagram depicting hypermethylation at specific CpG sites near the transcription start site, potentially inhibiting transcription factor binding and leading to *APCDD1* silencing. **(H)** Decitabine treatment reverses *APCDD1* silencing. ** P<0.01, *** P<0.001.

We systematically analyzed the expression of *APCDD1* and its epigenetic regulation in WT. Results showed that mRNA expression of *APCDD1* was significantly downregulated in the WT cell line WiT49 compared to human embryonic kidney 293T cells (**Figures 9A, B**). Similarly, *APCDD1* expression was markedly reduced in anaplastic WT samples (**Figure 9C**). Furthermore, the downregulation of *APCDD1* was strongly associated with hypermethylation of its promoter region (**Figure 9D**). Further analysis revealed that this low expression correlated negatively with high expression levels of both *DNMT3A* and *DNMT1* (**Figures 9E, F**), suggesting that DNA methylation mediated by these DNA methyltransferases is a key mechanism for APCDD1 silencing. Crucially, multiple CpG sites, including cg06825512 located near the transcription start site (TSS), exhibited a hypermethylated state in tumor tissues. This hypermethylation likely directly impedes transcription factor binding, collectively contributing to the transcriptional repression of *APCDD1* (**Figure 9G**). Functional validation experiments demonstrated that treating WiT49 cells with the DNA methyltransferase inhibitor decitabine led to a significant, dose-dependent upregulation of *APCDD1* mRNA levels (**Figure 9H**). This finding further confirms that *APCDD1* expression is under epigenetic control via DNA methylation. In summary, *APCDD1* is downregulated in WT due to hypermethylation of its TSS region. This low-expression signature may provide new insights for epigenetic research and identify potential therapeutic targets for WT.

### Leflunomide effectively suppresses malignant phenotypes of WT

**Figure 10:**
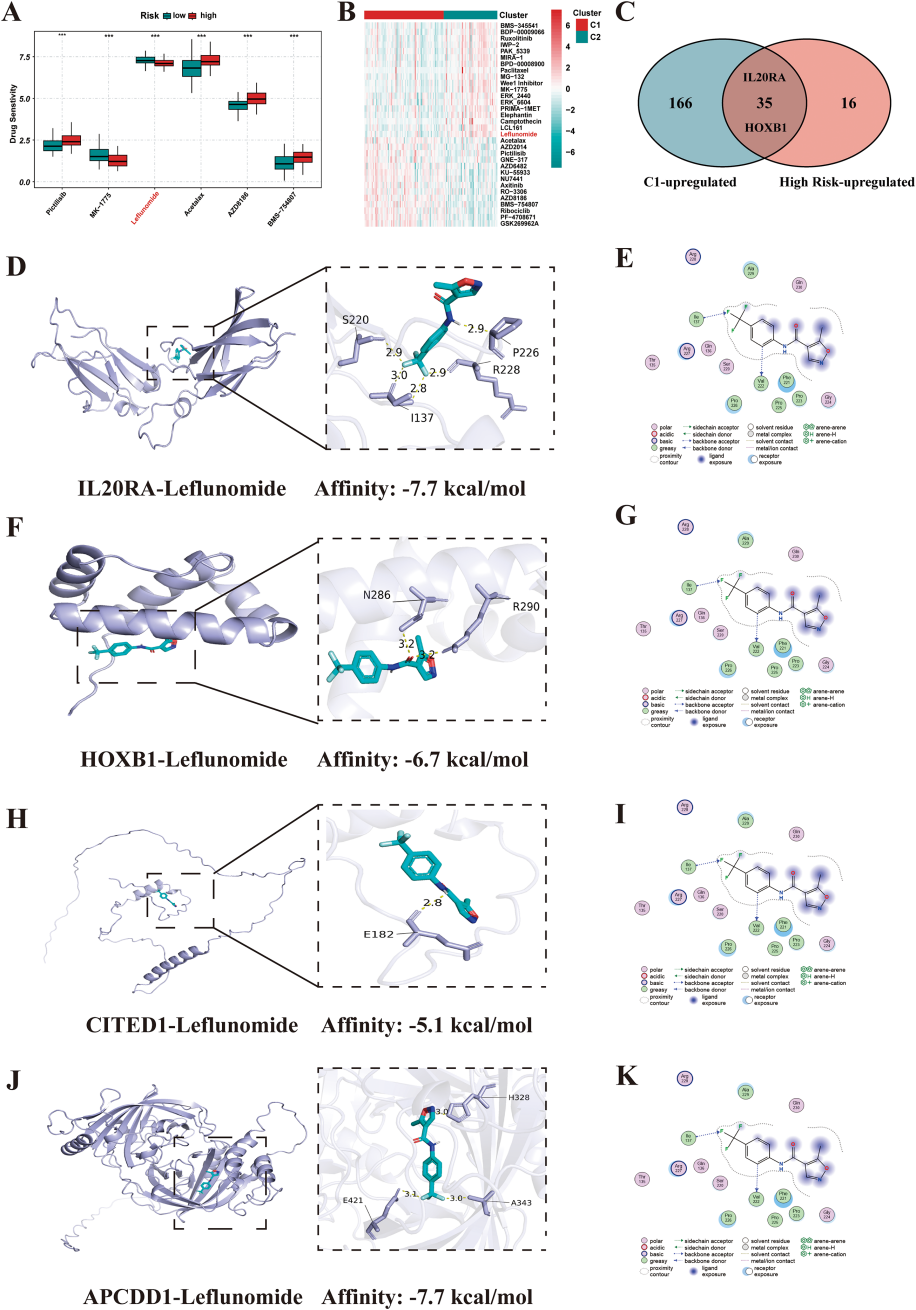
Computational identification of Leflunomide as a potential therapeutic agent for C1 subtype/High-risk Wilms tumor. Sensitivity analysis of multiple candidate drugs against Wilms tumor with High-risk/Low-risk status **(A)** and C1/C2 subtypes **(B)**. **(C)** The overlapping upregulated genes in both High-risk and the C1 subtype Wilms tumor. Molecular docking reveals strong binding affinity of Leflunomide to key proteins *IL20RA* **(D, E)**, *HOXB1* **(F, G)**, *CITED1* **(H, I)**, and *APCDD1* **(J, K)**.

Given the poor prognosis associated with the C1 subtype and high-risk WT patients, and considering the potential limitations of conventional chemotherapy, we screened and evaluated potential small-molecule drugs to explore more precise therapeutic strategies (**Supplementary Figure 3A**). Integrated analysis indicated that Leflunomide possessed relatively high therapeutic potential for the C1 subtype/high-risk WT (**Figures 10A, B**). Based on differential expression profiling, 35 genes consistently significantly overexpressed in both groups were identified. CMap database analysis revealed that Leflunomide could reverse the expression pattern of this gene set (**Figure 10C**, **Supplementary Figure 3B**). Molecular docking analysis further demonstrated high binding affinity between Leflunomide and the characteristically overexpressed protein *IL20RA* and *HOXB1* (**Figures 10D–G**). Concurrently, the stemness marker *CITED1* and the prognostic signature protein *APCDD1* also showed significant binding potential with Leflunomide (**Figures 10H–K**). These results suggest that Leflunomide may exert its effects by inhibiting *IL20RA*/*HOXB1* related pathways and modulating networks involving *CITED1* and *APCDD1*, providing molecular level evidence for its potential application in the C1 subtype and high-risk WT.

**Figure 11:**
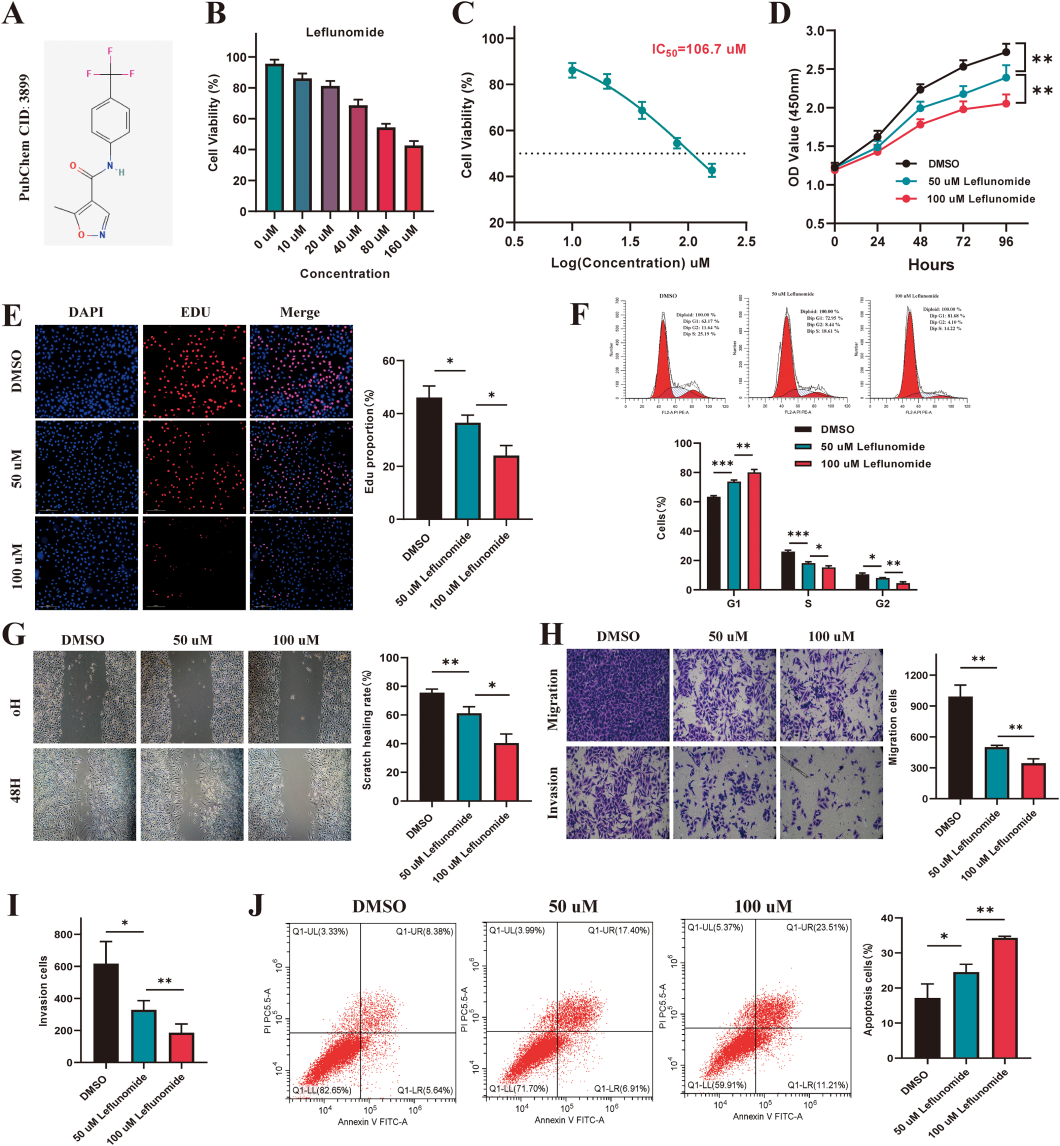
Leflunomide inhibits proliferation, migration, and invasion, and induces apoptosis in Wilms tumor. **(A)** Chemical structure of Leflunomide. Leflunomide concentration-dependently inhibits the viability of WiT49 cells **(B)**, with an IC50 of approximately 106 µM **(C)**. CCK-8 **(D)** and EdU **(E)** incorporation assays confirm the significant anti-proliferative effect of Leflunomide. **(F)** Cell cycle analysis shows that Leflunomide treatment induces G1 phase arrest and reduces the S phase population. Wound healing **(G)** and Transwell **(H, I)** assays demonstrate that Leflunomide effectively suppresses the migration and invasion capabilities of WiT49 cells in a concentration-dependent manner. **(J)** Flow cytometry analysis indicates that Leflunomide promotes apoptosis in WiT49 cells. * P<0.05, ** P<0.01, *** P<0.001.

As an approved dihydroorotate dehydrogenase inhibitor, Leflunomide blocks pyrimidine synthesis, inducing apoptosis and cell cycle arrest. It has demonstrated antitumor and anti-metastatic activity in various cancer models, highlighting its broad potential for “drug repurposing” (**Figure 11A**). *In vitro* assays in the WT cell line WiT49 confirmed that Leflunomide inhibits proliferation in a concentration dependent manner, with an IC50 of approximately 106 µM, indicating a potential anti-proliferative effect on WT cells (**Figures 11B, C**). CCK-8 and EdU assays substantiated the significant inhibition of proliferation (**Figures 11D, E**). Cell cycle analysis revealed G1 phase prolongation and S phase shortening (**Figure 11F**). Wound healing and Transwell assays both demonstrated Leflunomide’s ability to significantly inhibit the migration and invasion of WiT49 cells, with effects strengthening at higher concentrations (**Figures 11G–I**). Flow cytometry further indicated that it promotes WiT49 cells apoptosis (**Figure 11J**). In summary, Leflunomide exerts multidimensional effects in the C1 subtype and high-risk WT by inhibiting proliferation, suppressing migration and invasion, and inducing apoptosis.

## Discussion

With the rapid advancement of modern pediatric oncology, the treatment of WT still faces ongoing challenge. Specifically, a subset of cases exhibits characteristics such as refractoriness or a predisposition to relapse. Furthermore, the long-term toxic effects of existing chemotherapy modalities also constrain improvements in the quality of life for affected patients (4, 5, 8, 10). The key to overcoming the current therapeutic bottlenecks for this tumor lies in a deeper dissection of its molecular heterogeneity, to establish precise prognostic prediction systems and targeted intervention strategies. Identifying key molecular drivers of stemness in WT can not only optimize risk stratification but also provide a theoretical foundation for developing targeted therapies. Therefore, this study focused on the stemness characteristics of WT, aiming to systematically decipher its molecular mechanisms and provide new insights for achieving more precise risk stratification and therapeutic intervention in the clinic.

Driven by the objectives outlined above, this study systematically uncovered the molecular underpinnings of stemness features in WT through the integration of multi-omics data and in-depth bioinformatic analysis. Consequently, we constructed relevant molecular subtypes and a high-accuracy prognostic prediction signature. To our knowledge, this study provides a systematic elucidation of the expression regulatory mechanism and clinical significance of the *APCDD1* gene in WT. Furthermore, via a systematic drug screening approach, we identified and experimentally validated the potential therapeutic value of Leflunomide in targeting WT stemness characteristics. The principal innovation of this study lies in the translation of stemness characteristics, defined at the single-cell level, into a quantifiable metric applicable for clinical prognostic assessment. Specifically, grounded in the CytoTRACE algorithm, we established a novel stemness scoring system tailored for WT. This scoring system not only effectively addressed the inherent challenge of strong expression heterogeneity associated with traditional CSCs markers in solid tumors through bioinformatic means but, more importantly, it established for the first time a clear quantitative link between WT stemness features and clinical prognosis. This advancement successfully transforms the concept of tumor stemness, which has long been primarily a theoretical focus, into an objective, quantifiable indicator usable for clinical evaluation, thereby laying a solid foundation for future precision medicine practices in WT.

While the traditional histopathological classification of WT (favorable histology and anaplastic) provides a fundamental framework for clinical management, its inherent limitations are increasingly apparent (36, 37). This classification not only relies on subjective morphological assessment but, more critically, fails to reveal the molecular and functional heterogeneity within tumors, leading to significant prognostic variability among patients within the same subtype (38). To overcome this bottleneck, our study employed consensus clustering, a robust unsupervised machine learning method, to perform an objective molecular classification of WT based on differentially expressed SRGs profiles. The analysis clearly segregated WT into two subtypes (C1 and C2) with distinct biological characteristics. The C1 subtype exhibited a higher stemness score, which was strongly associated with inferior clinical outcomes. In-depth analysis revealed that the C1 subtype is characterized by a significantly immunosuppressive TME, featuring NK cell functional exhaustion, increased infiltration of regulatory T cells, and inhibited antigen presentation pathways. This finding indicated the co-existence of stemness features and an immunosuppressive TME, aligns with recent studies in other pediatric solid tumors, suggesting that combined strategies targeting tumor stemness and reversing immunosuppression may represent a crucial future direction. Furthermore, our study challenges the conventional view that high TMB is essential for triggering an effective anti-tumor immune response. In WT, a cancer typified by low overall TMB, our findings indicate that the intrinsic stemness features of the tumor cells themselves are likely the core driver shaping its unique immunosuppressive microenvironment. This insight has profound clinical implications. It suggests that for WT patients belonging to the C1 subtype, even though their TME is immunosuppressive, the use of immune checkpoint inhibitors (e.g., PD-1/PD-L1 inhibitors) alone might yield limited efficacy (39). This is because the root of immune suppression lies more in the non-mutational stemness program (40). Consequently, for this specific patient population, future efforts are urgently needed to develop novel combination therapies that can simultaneously target the core stemness pathways and remodel the tumor immune microenvironment, aiming to break through the current therapeutic limitations.

Molecular subtyping revealed significant inter-tumoral heterogeneity in WT. However, clinical decision-making urgently requires tools capable of directly assessing individual prognostic risk. Therefore, constructing a robust multi-gene prognostic signature serves as a bridge connecting biological insights with clinical precision medicine practice. The establishment of a prognostic prediction signature based on four risk genes marks a shift in WT prognosis assessment from traditional clinicopathological indicators toward molecular functional signatures. Using SHAP analysis, we quantified the contribution of each gene to the signature’s output. This confirmed *APCDD1* and *BICC1* as protective factors, potentially maintaining cellular differentiation status by regulating key developmental signaling pathways such as WNT (41, 42). In contrast, *CCL18* was identified as a risk factor, possibly promoting tumor progression by recruiting immunosuppressive cells (43, 44). More importantly, to explore the potential causality behind these associations, we further performed Mendelian Randomization analysis. The results provided genetic-level causal evidence supporting the protective roles of *APCDD1* and *BICC1*. This pattern of multi-gene synergy reflects the complexity of WT pathogenesis and progression, and also suggests that interventions targeting a single factor may be insufficient to achieve optimal therapeutic efficacy.

An in-depth investigation of the *APCDD1* gene represents another significant breakthrough of this study. *APCDD1* is an endogenous inhibitor of the Wnt/β-catenin signaling pathway (41). It directly binds to Wnt3A and the co-receptor LRP5, thereby blocking canonical Wnt signaling (41). Concurrently, its promoter contains TCF/LEF binding sites that can be activated by the β-catenin/TCF4 complex, forming a negative feedback loop (41). Interestingly, the role of *APCDD1* appears to be context-dependent across different cancers. In colorectal cancer, *APCDD1* expression is elevated, and its exogenous overexpression promotes cancer cell proliferation, migration, and invasion, while knockdown suppresses growth and induces apoptosis, suggesting an oncogenic function (45, 46). Conversely, in osteosarcoma, *APCDD1* expression is downregulated due to promoter hypermethylation, which relieves the inhibition on the Wnt/β-catenin pathway, thereby promoting EMT, enhancing invasion and metastasis, and potentially fostering tumor stemness (47). Given that the Wnt pathway is one of the core pathways maintaining the self-renewal and multi-directional differentiation of CSCs, *APCDD1*, as its endogenous inhibitor, could theoretically attenuate tumor stemness (21, 22, 48). In our study on WT, we found that *APCDD1* expression was significantly downregulated in tumor tissues and cell lines, and its low expression was closely associated with an unfavorable prognosis. Mechanistic studies revealed that hypermethylation of the *APCDD1* promoter region is the primary cause of its transcriptional silencing. Multiple CpG sites located near the transcription start site (e.g., cg06825512) exhibited a hypermethylated state in tumor tissues, which likely impedes transcription factor binding and consequently suppresses its expression. This finding was further supported by functional experiments, where decitabine treatment significantly upregulated *APCDD1* expression levels in WiT49 cells. From a functional perspective, as an antagonist of the WNT pathway, the downregulation of *APCDD1* may lead to aberrant activation of the β-catenin signaling pathway, thereby promoting the stemness maintenance of CSCs. Crucially, a two-sample Mendelian randomization analysis provided further evidence supporting a potential causal relationship between genetically determined *APCDD1* expression levels and WT risk, offering genetic support for its potential role as a therapeutic target for WT patients.

In drug screening research, leflunomide was found to exhibit significant antitumor sensitivity in the C1 subtype and high-risk WT. Further mechanistic studies suggest that leflunomide may exert its antitumor effects through multiple pathways. Molecular docking analysis revealed that the drug can bind with high affinity to multiple targets such as *IL20RA* and *HOXB1*, and can regulate genes related to tumor stemness including *CITED1* and *APCDD1*. As an inhibitor of dihydroorotate dehydrogenase, leflunomide can also induce cell cycle arrest and apoptosis by blocking the pyrimidine synthesis pathway (49). *In vitro* experiments further confirmed that the drug can inhibit the proliferation, migration, and invasion capabilities of WiT49 cells in a concentration-dependent manner. Particularly noteworthy is that leflunomide may possess unique advantages in targeting CSCs. Traditional chemotherapeutic drugs primarily act on rapidly proliferating tumor cells but have limited effects on most quiescent CSCs (22, 50). Leflunomide may interfere with the self-renewal capacity of CSCs by affecting developmental signaling pathways, thereby potentially fundamentally improving the treatment landscape for WT. However, targeting CSCs still faces numerous challenges, such as their high heterogeneity and plasticity, which may lead to resistance to monotherapy. Therefore, future research could explore combination strategies of leflunomide with other drugs. For example, combining it with demethylating agents may synergistically inhibit the WNT signaling pathway by upregulating *APCDD1* expression, thereby enhancing efficacy.

In fact, the anti-tumor potential of leflunomide is not limited to WT. In models of prolactinoma, neuroblastoma, melanoma, and pancreatic cancer, it has demonstrated tumor-suppressive effects either as monotherapy or in combination with targeted/chemotherapeutic agents by inhibiting *DHODH* and depleting pyrimidines (51–53). Recent studies have demonstrated that without diminishing the efficacy of PD-1 inhibitors, leflunomide can significantly reduce immune-related cardiotoxicity through the “gut microbiota-indolepropionic acid-AHR” axis, suggesting its dual value of “enhancing efficacy and reducing toxicity” in cancer immunocombination therapy (54). Overall, the discovery of leflunomide’s effects holds significant translational medical value. As an FDA approved drug, its pharmacokinetic and safety data are relatively clear, facilitating “drug repurposing.” Combined with its accumulated clinical application experience in treating rheumatoid arthritis, repurposing it for pediatric WT treatment is expected to reduce development risks and shorten the R&D cycle (55).

This study proposes a preliminarily discovery framework for a WT molecular subtyping and prognostic prediction system based on tumor stemness characteristics, achieving breakthroughs in mechanism exploration and drug discovery. However, several aspects require further refinement. Notably, the current therapeutic validation is primarily based on computational analysis and *in vitro* assays using a single cell line. Future research should focus on three key areas: First, we will expand the experimental validation by employing multiple Wilms tumor cell lines and establishing standardized patient-derived organoid models to confirm the reproducibility and broader relevance of our findings. Second, deepening the investigation into *APCDD1* methylation mechanisms regulating stemness features, utilizing gene editing technologies to validate causal relationships, and systematically analyzing Leflunomide’s targeting pathways for cancer stem cells through *in vivo* experiments. Third, promoting technical simplification and standardization by developing streamlined molecular subtyping protocols suitable for routine pathological testing to reduce clinical application barriers. By overcoming these technical bottlenecks, we can potentially shift the WT diagnosis and treatment paradigm from traditional morphological classification to molecular function-driven precision medicine, ultimately improving patient outcomes.

## Conclusion

In summary, this study deciphers the stemness-driven molecular landscape of WT, offering a preliminary discovery framework that includes relevant molecular subtypes and prognostic risk signature. We highlight *APCDD1* as a key epigenetically silenced protector and propose Leflunomide as a promising therapeutic candidate for WT patients. These insights pave the way for transitioning WT diagnosis and treatment from histology-based to molecular function-guided precision medicine, with the potential to improve risk stratification and therapeutic outcomes for pediatric patients.

## Data Availability

The original contributions presented in the study are included in the article/**Supplementary Material**. Further inquiries can be directed to the corresponding authors.
