# Supplementary figures and images for: Multi-omics analysis identifies stemness-driven molecular subtypes, prognostic signature, epigenetic target APCDD1, and drug candidate Leflunomide in Wilms tumor
Source: Front Oncol. 2026 May 25;16:1775326. doi: 10.3389/fonc.2026.1775326 (PMC13244880; doi:10.3389/fonc.2026.1775326)

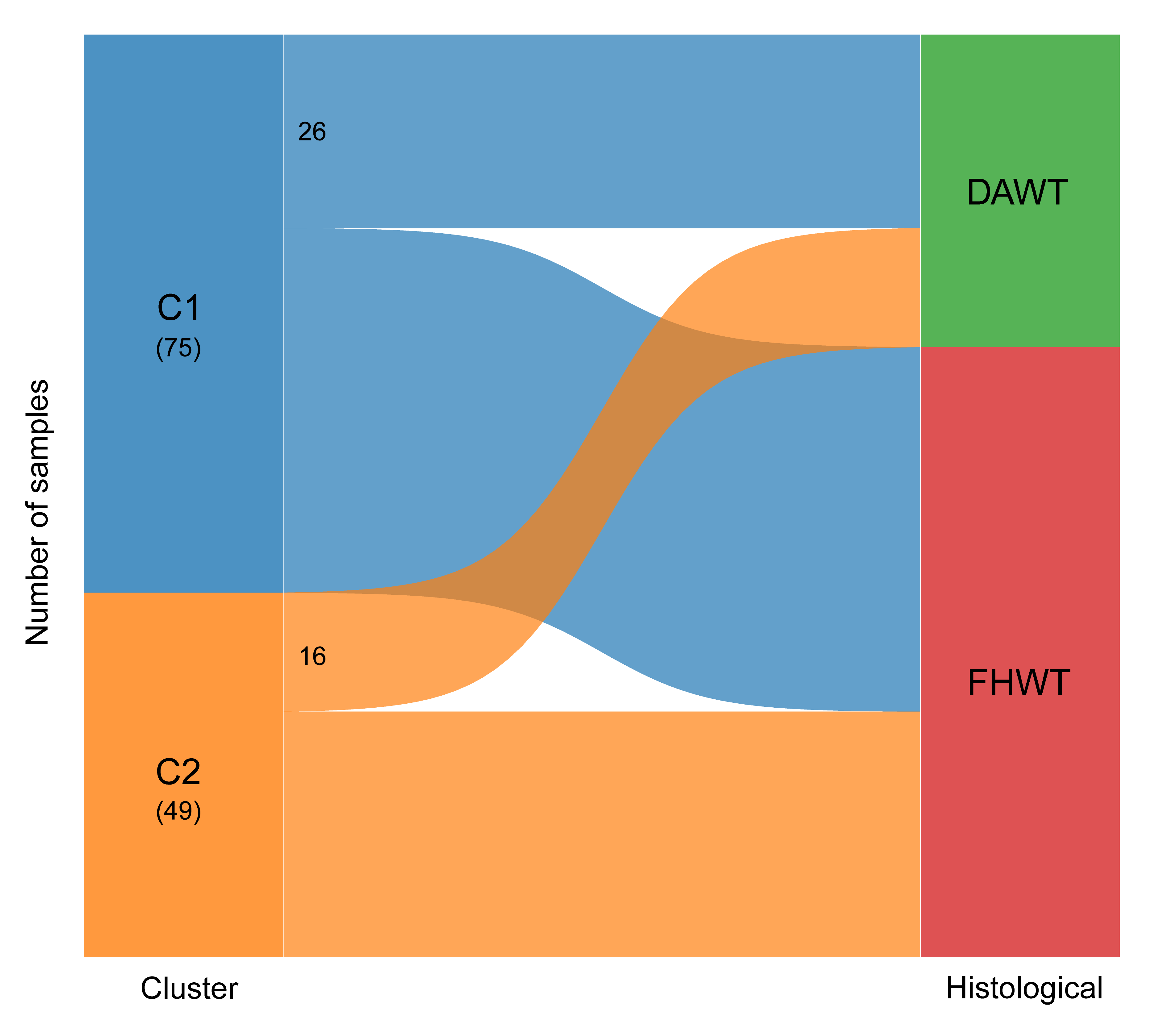

Supplement: Supplementary file 2 [file Image2.tif]

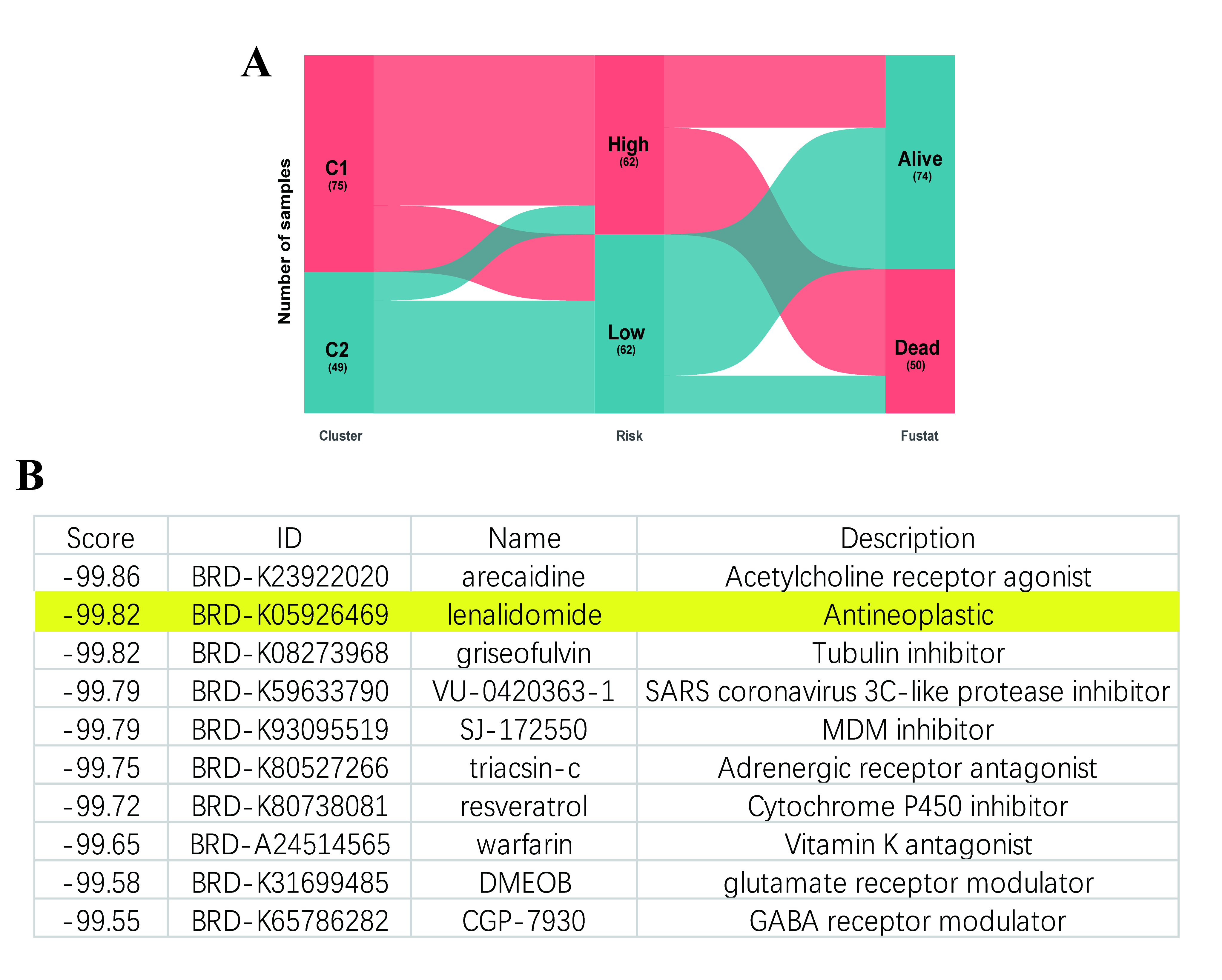

Supplement: Supplementary file 3 [file Image3.tif]
